# Non-cultivated plants present a season-long route of pesticide exposure for honey bees

**DOI:** 10.1038/ncomms11629

**Published:** 2016-05-31

**Authors:** Elizabeth Y. Long, Christian H. Krupke

**Affiliations:** 1 Department of Entomology, The Ohio State University, OARDC, 1680 Madison Ave, Wooster, Ohio 44691, USA; 2 Department of Entomology, Purdue University, 901 West State Street, West Lafayette, Indiana 47907, USA

## Abstract

Recent efforts to evaluate the contribution of neonicotinoid insecticides to worldwide pollinator declines have focused on honey bees and the chronic levels of exposure experienced when foraging on crops grown from neonicotinoid-treated seeds. However, few studies address non-crop plants as a potential route of pollinator exposure to neonicotinoid and other insecticides. Here we show that pollen collected by honey bee foragers in maize- and soybean-dominated landscapes is contaminated throughout the growing season with multiple agricultural pesticides, including the neonicotinoids used as seed treatments. Notably, however, the highest levels of contamination in pollen are pyrethroid insecticides targeting mosquitoes and other nuisance pests. Furthermore, pollen from crop plants represents only a tiny fraction of the total diversity of pollen resources used by honey bees in these landscapes, with the principle sources of pollen originating from non-cultivated plants. These findings provide fundamental information about the foraging habits of honey bees in these landscapes.

## Abstract (web summary)

The extent to which non-crop plants may be contaminated by insecticides is not known. Here, the authors show that pollen collected by honey bees living in areas of intensive maize production is contaminated by a wide range of pesticides throughout the growing season, with the principle pollen source being non-crop plants.

## Graphical abstract

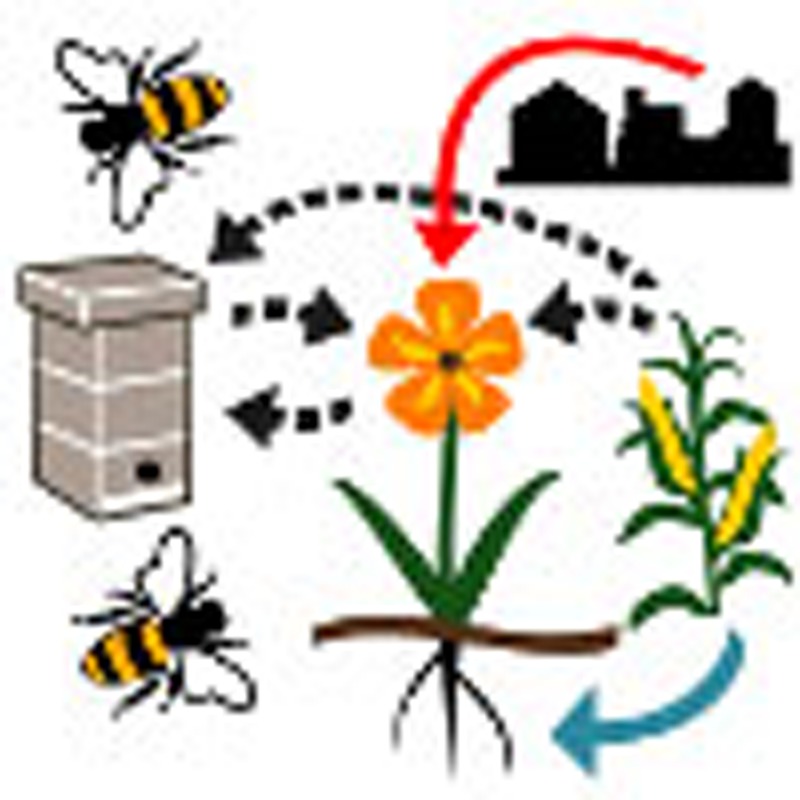

The declines of honey bees and other pollinators have been at the forefront of recent scientific publications and popular press. Accumulating evidence suggests that no single stressor alone is responsible for declines. Rather, it is probably a combination of abiotic and biotic factors acting in synchrony, to have a negative impact on pollinator populations^1^,^2^,^3^,^4^,^5^,^6^. Pesticides are stressors that have received considerable attention, and among these no single class has received more recent attention than the neonicotinoids^7^,^8^,^9^,^10^,^11^,^12^,^13^,^14^. These insecticides are acutely toxic to honey bees, environmentally persistent and mobile in the environment^15^,^16^,^17^,^18^,^19^. They are also among the most widely used insecticide classes in grain and oilseed crops: in the United States, 36.6 million hectares of maize and 33.8 million hectares of soybeans were planted in 2014 (ref. 20), with 79–100% and 34–44% of these seeds, respectively, treated before planting with a formulation of neonicotinoid insecticides (typically thiamethoxam or its metabolite clothianidin)^21^. Although it is known that neonicotinoids can and do move from crop fields during planting, as dust, and afterwards in ground and surface water, the extent of this contamination in the environment throughout the foraging season of honey bees has only recently begun to be quantified^16^,^19^,^22^,^23^,^24^,^25^,^26^; therefore, this presents a relatively unexplored route for pollinator exposure to this pesticide class. Moreover, the presence of other, potentially synergistic, pesticides encountered in honey bee food resources has seldom been examined^27^ and most published work in this area tends to focus exclusively on the occurrence of neonicotinoid insecticides in pollen or nectar resources^7^,^28^,^29^,^30^,^31^,^32^,^33^; however, exposures of mobile insects such as pollinators to a single pesticide rarely occur in field situations^34^. Given the vast acreages devoted to these crops and concerns about worldwide pollinator decline, we initiated this work to describe both how (that is, plant species) and when (that is, time in season), pollen-foraging honey bees are exposed to a range of pesticides in agricultural landscapes, with an eye towards clarifying potential high-risk compounds and identifying common combinations of pesticides encountered in field environments.

Pollen collected by honey bees was collected, identified and screened for agricultural pesticides over a period of 16 weeks in 2011. We initiated this study after all planting of treated seeds in our study was completed, to minimize dust from pneumatic planters as a direct source of pesticide residues. Two Langstroth hives were placed at each of three sites: (1) an open meadow with wildflowers, woody shrubs and trees present (non-agricultural site), (2) the border of a maize field planted with seeds treated with the neonicotinoid clothianidin and three fungicides, and (3) the border of a maize field planted with seeds that received no pesticide treatment. To characterize the landscape surrounding honey bee hives, proportions of different land covers within a 2-km radius around each of the three sites were extracted from the 2012 Cropland Data Layer produced by the US National Agricultural Statistics Service using QGIS (QGIS Development Team 2015). Honey bee colonies remained at each site for the length of the growing season and foraging bees had free access to pollen from all crop and non-crop species in the vicinity. Analysis of forager-collected pollen revealed that in all cases honey bees foraged primarily on non-cultivated plants and residues of multiple pesticides were found throughout the season. Although a variety of agricultural pesticides were found at all sites, the contaminants likely to provide the greatest hazard to honey bees in our study were non-agricultural pyrethroid insecticides targeting nuisance pests such as mosquitoes.

## Results

### Foraging on pesticide-contaminated non-cultivated plants

Pollen collected by honey bees was consistently contaminated with pesticides throughout the 16-week period and the overwhelming majority of pollen was collected from non-cultivated plants. Pesticide residue analyses of bee-collected pollen revealed contamination by up to 32 different pesticides spanning 9 chemical classes (Table 1). The most common pesticide types detected in pollen samples across all sites were fungicides and herbicides. Honey bees visited a diverse assemblage of flowering plants, collecting pollen from up to 30 plant families during the 16-week sampling period (Table 2). Regardless of the location of honey bee colonies, pollen loads were dominated by the Fabaceae; a plant family that includes both wild and cultivated species such as clovers, alfalfa and soybeans.

### Pesticides in pollen collected in non-agricultural areas

**Figure 1:**
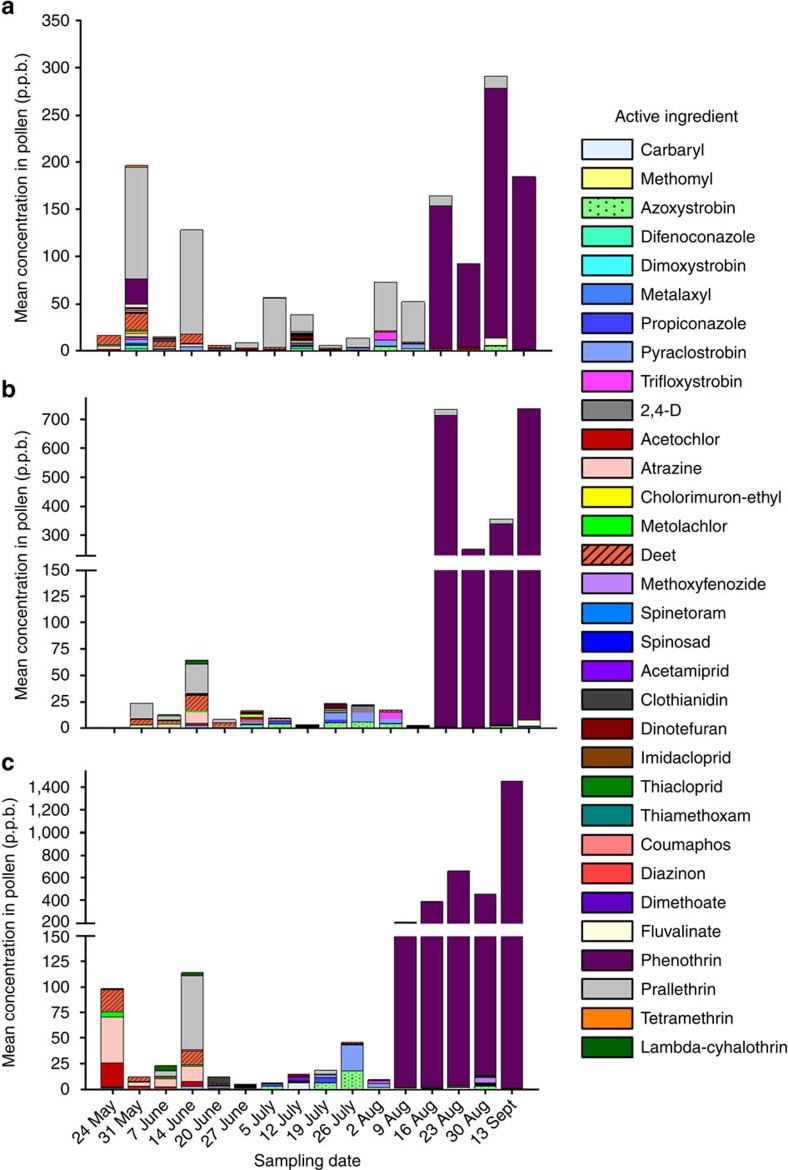
The mean concentration of pesticide-active ingredients detected in pollen collected by honey bees from three sites that vary in surrounding land-use types. (**a**) Non-agricultural area. (**b**) Adjacent to untreated maize field. (**c**) Adjacent to neonicotinoid-treated maize field. p.p.b., parts per billion.

Analysis of the pollen collected by honey bees in the non-agricultural area revealed contamination with 29 pesticides, the most common of which were the fungicides azoxystrobin and trifloxystrobin (93.3 and 63.3% of samples), the herbicide metolachlor (83.3%) and the pyrethroid insecticides prallethrin and phenothrin (46.7 and 30% of samples) (Table 1). Carbamate, neonicotinoid and organophosphorus insecticides were also detected, although less frequently in 3–16.7% of pollen samples. Mean pesticide concentrations in pollen over the sampling period varied from 6–317 p.p.b., with no clear seasonal pattern (Fig. 1a). The highest mean concentrations of pesticides in pollen occurred during August and September, and were driven largely by the pyrethroid insecticide phenothrin. During these sampling periods, pollen loads were dominated by the family Brassicaceae (mustards or crucifers). Pollen from plants in the Fabaceae (legumes, peas and beans) were present in highest quantity over the course of the season at the non-agricultural site; however, the greatest proportion of pollen collected by bees during any one sampling period was from the Brassicaceae and likely from wild mustard, *Sinapsis arvensis*, an insect-pollinated annual species that was particularly common at that site. Although pollen from non-cultivated plants dominated honey bee loads over the course of our experiment, foragers did occasionally visit soybean (*Glycine max*) and less frequently maize (*Zea mays*) to collect pollen, despite the distance from the meadow to surrounding crop fields (Table 2). Honey bees at this site collected 0.4% of their pollen from maize plants and this was restricted to the week of 19 July, while up to 7.9% of their pollen was collected from soybean plants over the course of 5 weeks (2–30 August).

### Pesticides in pollen collected adjacent to untreated maize

We detected 31 different pesticide residues in pollen from honey bee colonies placed at the maize field grown from untreated seed. As was the case at the non-agricultural site, the most common pesticides detected were the fungicides azoxystrobin and trifloxystrobin (87.5 and 62.5% of samples), as well as the herbicides metolachlor and atrazine (75 and 54% of samples, respectively) (Table 1). Of the neonicotinoid insecticides detected, thiamethoxam was present most frequently in 33% of pollen samples. Once again the carbamates, neonicotinoids and organophosphates were generally less prevalent in pollen than the fungicides and herbicides at this site, with 4–33% of pollen samples containing these residues. Mean levels of pesticide contamination varied over the sampling period from 3–736 p.p.b., with no clear seasonal pattern, except that concentrations were higher later in the season than at any other period (Fig. 1b). The highest concentrations occurred mid-August and mid-September, and were again driven by spikes of the pyrethroid phenothrin. These periods corresponded with large proportions of common ragweed pollen *Ambrosia artemisiifolia* and two other pollen species, one unknown and another unidentified member of the Asteraceae. Although maize and soybean pollen was readily available in the vicinity during mid-summer, the majority of pollen collected by honey bees throughout the season came from non-cultivated plants (Table 2). Foragers collected no more than 17.6 and 6.3% of their pollen from maize and soybean plants, respectively, during any given week, and this was restricted to a 5- to 6-week period (12 July–23 August for maize and 5 July–9 August for soybean).

### Pesticides in pollen collected adjacent to treated maize

Pollen collected by honey bees at the maize field grown from pesticide-treated seed was contaminated with 28 pesticide residues, a number similar to the other sites. However, overall mean concentrations of active ingredients were significantly higher, up to 1,453 p.p.b. (Fig. 1c). Fungicides (azoxystrobin and trifloxystrobin) and herbicides (metolachlor, atrazine and acetochlor) were again the most frequent contaminants of bee-collected pollen (34–87.5% of samples), followed by the active ingredients phenothrin (pyrethroid), acetamiprid (neonicotinoid) and carbaryl (carbamate), which were present in 25–28% of pollen samples (Table 1). Overall, concentrations of contaminants were highest late in the season and the highest concentration of active ingredients occurred during the last sampling period (13 September), driven almost exclusively by contamination with the pyrethroid insecticide phenothrin. The phenothrin peak in honey bee pollen coincided with large proportions of pollen from two plants, one unknown and the other an unidentified member of the Asteraceae. Consistent with the other locations, pollen collected by honey bees at the pesticide-treated maize site was dominated by non-cultivated plants (Table 2). Foraging bees collected no more than 21.1 and 4.4% of their pollen from maize and soybean plants, respectively, during any given sampling period and this was restricted to a 5- to 6-week period (12 July–30 August for maize and 5 July–9 August for soybean).

Finally, we gathered honey bee toxicity data from the International Union of Pure and Applied Chemistry Pesticide Properties Database (http://sitem.herts.ac.uk/aeru/iupac), the US Environmental Protection Agency, the National Pesticide Information Center (http://npic.orst.edu/ingred/aifact.html#ecotox) and the open literature^35^,^36^, to interpret our results from the perspective of a risk assessment. Toxicity data were used in combination with the frequency and concentrations of pesticides we detected in pollen, to calculate the risk (probability) that 50% of honey bees that ingest or contact the contaminated pollen in our study would experience mortality (Tables 3, 4, 5).

## Discussion

This study demonstrates that the primary pollen resources used by honey bees living near field crop landscapes are non-cultivated plants, and that these pollen sources are frequently contaminated with a range of pesticides^37^,^38^. Despite collecting only small amounts of crop pollen throughout most of the season, the pollen collected by honey bees at all three sites exhibited contamination with multiple pesticides during the entire sampling period. Surprisingly, although the pesticides used in agricultural production were consistently present as contaminants of honey bee-collected pollen, they were not the contaminants present in highest concentrations. Furthermore, this finding was consistent across sites, even though the surrounding land use types differed between the non-agricultural area and the two maize sites (Table 6).

Our study area is representative of a large portion of the United States that is characterized by intensive production of the top two crops, by land area, grown in the country—maize and soybeans^37^,^38^. Both of our maize sites reflect that a majority of the surrounding area is dedicated to the production of these two crops (Table 6). Maize and soybeans are commonly treated with neonicotinoids before planting, raising concerns about pollen from these crops as an exposure route for honey bees and other pollinators^21^. Honey bee-collected pollen from crops grown from neonicotinoid-treated seeds has been investigated previously as a source of exposure to this class of insecticides^7^,^8^,^32^,^33^,^39^,^40^ and recent work in the United Kingdom has detected neonicotinoid residues in pollen of wildflowers growing in areas where treated oilseed rape was sown^28^. The frequency and variety of neonicotinoids and other pesticides found in our analyses of diverse pollen sources, including a wide range of wildflower pollen, is striking given an environment that is dominated by large monocultures of two plant species. Given that this work was performed over a single season and across few sites in a limited area, we hesitate to generalize our findings across a wide range of systems. Furthermore, it was beyond the scope of our study to evaluate pesticide concentrations in each pollen species individually; thus, we cannot rule out the possibility that very low amounts of crop pollen may have contributed high concentrations of pesticide residues during some sampling periods.

The pesticide concentrations we documented in pollen samples spanned levels that may have a range of effects from sub-lethal to acutely toxic^41^. Although the majority of studies reporting concentrations of pesticide active ingredients in honey bee matrices generally focus on the concentration of contaminants relative to the honey bee median lethal dose (LD_50_) as an indicator of risk posed by a particular pesticide, a more comprehensive evaluation of pesticide risk to honey bees has been proposed. This approach incorporates the frequency of detection of pesticides in relevant matrices, namely pollen and nectar, to gain a more realistic picture of the risk of mortality posed to bees that are exposed to varying concentrations of pesticides with a range of toxicities^34^. Our data are ideally suited to this type of analysis. Following this approach, we calculated the oral and contact risks posed to honey bees based on (1) pesticide residue loads in pollen, (2) the detection frequency of each pesticide in pollen samples and (3) reported honey bee oral and contact LD_50_ values. Oral risk was calculated based on the amount of pollen a honey bee consumes in its lifetime during its role as a nurse bee (65 mg)^42^, while contact risk was based on contact with 1 g of pollen each day^34^. Performing these calculations with our data (Tables 3, 4, 5) highlights the importance of simultaneously considering both the toxicity (LD_50_) and the frequency of encountering pesticide active ingredients in pollen. Nine active ingredients of the 32 detected in pollen samples were relevant in terms of posing a low (risk values=0.1–1%, 2–4 chemicals at each site), moderate (1–5%, 2–3 chemicals at each site) or high risk of mortality (>5%, 3–4 chemicals at each site) to 50% of honey bees exposed to the chemicals in collected pollen^34^ throughout the season. A key finding of our pesticide risk assessment was that pesticides used in agricultural production, although important, were not the contaminants that posed the highest risks in honey bee-collected pollen. Rather the pyrethroids phenothrin and prallethrin, used mainly as dusts or sprays to manage mosquitoes, fleas and ticks^43^,^44^, stood out as posing exceptionally high risks to honey bees throughout the sampling period and across all sites, with risk values consistently >5% (Tables 3, 4, 5). The neonicotinoid clothianidin, used as a maize seed treatment^21^ and a breakdown product of the neonicotinoid thiamethoxam, which is also used as a maize and soybean seed treatment^21^, posed a high seasonal risk to honey bees located adjacent to the pesticide-treated maize field, with a cumulative seasonal risk value of 9.49% (Table 5). We were unable to identify the two plant species that dominated honey bee pollen loads during the weeks when the risks posed by phenothrin and prallethrin were highest; however, a contributor to the high seasonal risk (>5%) posed by clothianidin at the pesticide-treated maize site was probably pollen from the clothianidin-treated maize, which initiated anthesis (pollen shed) beginning early in July, making maize pollen readily available for collection by honey bees. We observed maize pollen in honey bee pollen loads in highest proportions during the weeks of 16–23 August (Table 5). Although the key pollen source visited by honey bees was unclear during the week of 20 June when the weekly risk posed by clothianidin was greatest, we can rule out the treated maize as a source, as this early date is in advance of maize anthesis in our area. The neonicotinoid thiamethoxam posed a high seasonal risk of mortality to honey bees located adjacent to the untreated maize field, yet again we can only speculate as to the source of the contaminated pollen, although it is not likely to be crop pollen. Wind-erodible surface soils have recently been reported to contain *ca*. 60 p.p.b. of neonicotinoid residues and may contribute to our results, as these dusts land on flowers that honey bees frequent^26^.

Our results also reflect that active ingredients that exhibit low toxicity may yet pose risks to honey bees if they are frequent contaminants of pollen throughout the season (that is, posing chronic risk). Conversely, highly toxic pesticides may pose relevant risk levels even if they are present only sporadically in pollen resources. These scenarios are especially important to consider in cases when two or more pesticides that exhibit synergy are detected simultaneously in pollen resources. We therefore calculated the potential risks posed by the frequency of synergistic mixtures between the fungicide propiconazole and certain neonicotinoids^45^, as well as with the pyrethroid *λ*-cyhalothrin^46^ in pollen samples. Although propiconazole alone did not pose relevant weekly or seasonal risks to honey bees at any of the three sites, its presence in pollen samples contributed to synergistic risks with other insecticides that pose a high risk of mortality to honey bees (risk values >5%), most notably a pyrethroid often used as a foliar treatment in both maize and soybeans, *λ*-cyhalothrin, at all three sites (Tables 3, 4, 5). Although the concentrations of pesticide residues in bee-collected pollen may change following processing and storage in colonies, the residues we detected in bee-collected pollen, at a minimum, present a season-long and ubiquitous (that is, across plant taxa) source of pesticide exposure for honey bees foraging in agriculture-dominated landscapes. Several studies have documented contamination of honey bee pollen with various pesticides^7^,^8^,^27^,^28^,^30^,^31^,^32^,^33^; however, these studies were either focused primarily in non-field crops settings, focus only on a single crop and over shorter time intervals, or documented substantially lower concentrations of the pesticides we detected.

Given the nature of our study, whereby we allowed honey bees to visit flowering resources freely as they bloomed throughout the season, it is impossible to definitively determine the main source of pesticide contamination at any period. The frequent detection of the fungicides azoxystrobin and trifloxystrobin is probably the result of residual amounts of these active ingredients remaining in the soil of surrounding crop fields, given that these fungicides are common ingredients in the seed treatment formulations applied to maize seeds before planting. Coumaphos is a common miticide that is frequently used in bee-keeping, which probably accounts for its presence in honey bee pollen at all sites. Given that we initiated our study late in the spring when the planting of seed-treated crops in the region was complete, the detection of thiamethoxam and clothianidin in pollen samples at all sites was unlikely to be due solely to drift from planter dust (during the planting of treated seeds) landing directly on blooming flowers. Rather, it is more likely due to residual seed treatment compounds present in soils^26^,^47^, or neonicotinoid-contaminated dusts landing on soils, where they are available for uptake by non-target plants and subsequently available to honey bees in pollen later in the season. Furthermore, the neonicotinoids we found are not generally applied as foliar sprays in our study system^21^, are highly water soluble^47^ and are prone to breakdown under ultraviolet light^48^, the latter making planter dusts on leaf surfaces a less probable exposure route later in season than movement via water from contaminated soils through plant tissues. High levels of prallethrin and phenothrin, two pyrethroid insecticides detected near the end of the sampling period, was a surprising finding. These products are not commonly used as agricultural pesticides, but are frequently used in and around municipalities for the management of mosquitoes, fleas and ticks^43^,^44^. The presence of N,N-Diethyl-meta-tolumide (DEET) in 100% of pollen samples was also surprising. Although it has been detected in surface and waste water^49^, it is a nonpolar compound that does not dissolve in water^50^, thus making it unlikely to move systemically into pollen from contaminated water in the environment. We speculate that this nonpolar compound may have been contacted by foraging honey bees in another way (that is, not on flowers) and transferred to wax, which is also nonpolar and a matrix that honey bees contact frequently within the hive. Taken together, these results suggest that an overemphasis on agricultural systems and the pesticides used there may fail to identify key sources of risk for pollinators, and furthermore that overall levels of pesticide exposure for honey bees living in areas dominated by annual crops such as maize, soybeans, rapeseed and wheat may be considerably higher than suggested by studies that have restricted analyses to neonicotinoid insecticides or the treated crop and its flowering period. The ubiquity of pesticide exposure in pollen throughout the season casts doubt on the viability of bee-friendly habitats adjacent to crop fields^51^ and instead suggests these habitats could act as putative ‘trap crops' acquiring multiple pesticides in the form of dust or spray drift, which may settle on and contaminate blooming flowers or, in the case of systemic chemicals, contaminate water with active ingredients that are subsequently taken up by plants and expressed in pollen^16^,^17^,^18^. This is of particular concern in light of recent work demonstrating that honey bees and bumble bees show no aversion to neonicotinoids presented in food^52^,^53^, and furthermore that these insecticides can impair honey bee immune responses to pathogens^54^. Moreover, the presence of multiple pesticides co-occurring in all samples is important in light of research that has demonstrated synergy, in terms of toxicity, for both honey bees and bumble bees, specifically between the fungicide propiconazole and the neonicotinoid insecticides acetamiprid and thiacloprid^45^, and the pyrethroid *λ*-cyhalothrin^46^ for honey bees, and between the neonicotinoid imidacloprid and *λ*-cyhalothrin for bumble bees^55^. We found combinations of these pesticides co-occurring in our samples throughout the season. In addition, fungicides found in our study, such as azoxystrobin, may contribute to the increased susceptibility of honey bees to infection by the gut pathogen *Nosema ceranae*^4^ and organophosphorus miticides such as coumaphos can interact with the neonicotinoid imidacloprid to affect odour learning in honey bees^56^. Strategies to mitigate the key pesticide risks identified above for honey bees and other pollinators will require concerted efforts to evaluate pest management practices not only in agricultural areas but in urban areas as well, with an eye towards the likelihood of pollinator exposure to pesticides in a given environment. Although the bulk of the current research and regulatory focus is on neonicotinoid insecticides used in agricultural crops, our work demonstrates that this approach is unlikely to yield the more substantial benefits that could be realized by incorporating risks posed by urban areas, in particular as the interface between urban and rural environments becomes more ambiguous. In addition, we must consider the exposure risks faced by pollinators in these two environments in terms of both the often-concurrent hazards posed by chemicals that are acutely toxic, but present transiently in the environment of pollinators (that is, pyrethroids), along with those that pose sublethal risks, because they are present consistently in the environment, at low concentrations, and for extended periods of time (that is, neonicotinoids).

These findings provide guidance in filling crucial gaps in our current knowledge of the plants that honey bees preferentially visit (and potentially require) in landscapes that are dominated by monocultures of maize and soybean. For example, bees from all colonies in our study collected the vast majority of their pollen from non-crop plants even when pollen-producing field crops were abundant in the landscape, suggesting that pollen from these crops is not a preferred food source. Our work demonstrates that the pest-management practices employed both within crop fields and beyond have implications for honey bee and other pollinator populations in the area, as both urban and agricultural pesticides were relatively common in all types of pollen throughout the season. Finally, it is critical to point out that although we used honey bees as pollen collectors in this experiment, it is likely to be that a wider range of pollen-feeding animals, including many wild bees and other non-target invertebrates are also exposed to the same suite of pesticides^8^,^10^,^57^ as they forage in and near agricultural fields.

## Methods

### Honey bee colonies and pollen identification

We selected six standard, or Langstroth, hives from the Purdue Honey Bee Lab (West Lafayette, IN) for inclusion in this study. Each consisted of two standard depth boxes with ten frames in each. Before the study, in early May of 2011, colonies were examined by pulling frames, ensuring that all colonies were queenright and of approximately equal strength. Sister queens from the same breeding line were used to ensure uniform genetic makeup of bees among treatments. Hives were then randomly assigned to each treatment. A bottom pollen trap (M000682, Dadant and Sons Hamilton, IL) was placed in each hive and hives were placed on top of two 10 × 10 cm wooden blocks, ∼60 cm in length, to minimize contact with the ground. Bottom pollen traps are placed at the base (entrance) of hives and contain a metal screen with circular openings just large enough to allow honey bees to enter. Foragers with full pollen loads are unable to fit through these openings and as they attempt to move through pollen falls off and accumulates in a tray below. Vegetation was trimmed around the base of hives so the entrance was not blocked. All hives were placed on lands owned by Purdue University. At the agricultural sites, hives were placed 3 m from the field margin with the opening facing the maize field. The maize fields were ∼3 km apart. The non-agricultural site was located ∼12 km from the treated and untreated maize fields, but because of the intensive production of maize and soybeans in our study area (Tippecanoe County, Indiana: ∼77,000 hectares in 2011)^58^, the non-agricultural site was in relatively close proximity (∼0.8 km) to agricultural fields where field crops were planted. The hives were placed on 17 May, 2011, a few hours after fields were sown. The same maize hybrid (Select Seeds 4980, Camden, IN) was used in both maize fields and sown at a rate of 79,040 kernels per hectare. Each field was ∼1.5 hectares in size. Treated maize consisted of the following commercially available seed treatment package: 3.0 ml of the fungicide metalaxyl per 45.4 kg of seed, 2.5 ml of the fungicide ipconazole per 45.4 kg, 9.5 ml of the fungicide trifloxystrobin per 45.4 kg of seed and 166.8 ml of the insecticide clothianidin per 80,000 kernels; untreated maize was planted as naked seed with no pesticides. Following initial placement, hives were opened once each month to locate the queen and ensure that bees had sufficient space. An additional box and frames were added ∼1 month after initial placement in the field. No other measurements of colony health were made during the experiment. For 16 weeks (24 May–13 Sept 2011), honey bee pollen was collected from the pollen trap and samples were stored at −20 °C for identification and pesticide residue analysis. Pollen from the two hives at each site was pooled by date, mixed well and a 2-g sub-sample of this mixture was used for pollen identification. The pollen pellets in each 2 g sub-sample were dissolved in water, agitated and homogenized, to ensure thorough mixing. Twenty microlitres of the pollen+water mixture was dried on a standard microscope slide, dyed with a fuchsin gelatin–glycerin mix that was melted over the dried 20 μl aliquot and sealed with paraffin and a coverslip. A representative slide of pollen granules was prepared for each date and each site following this process, and identification of pollen was conducted by randomly sampling 200–300 pollen grains on each microscope slide and comparing with a pollen reference library maintained by the Centre de Recherche en Sciences animales de Deschambault in Québec, Canada.

### Pesticide extraction and quantification

Pesticide residues were extracted from honey bee pollen using a modified QuEChERS method^59^. A total of 15 g of pollen from each hive, on each sample date, was homogenized and separated into three 50-ml centrifuge tubes and extractions were performed on three replicate pollen samples of 5 g each. Sufficient levels of honey bee pollen were not available for collection due to disturbance of the hives by vertebrate predators (probably striped skunks, *Mephitis mephitis*) on the following sample dates: Hive 1A (26 July), Hive 1B (24 May), Hive 2A (24 May, 2 August through 13 September) and Hive 2B (24 May). These ten sampling points were excluded from calculations of seasonal pesticide prevalence in pollen samples. Therefore, over the 16-week sampling period a total of 30 pollen samples were collected from colonies at the non-agricultural area, 24 samples from the colonies adjacent to the untreated maize field and 32 samples from the colonies adjacent to the neonicotinoid-treated maize field, for a grand total of 86 pollen samples across all sites. We added 30 ml of extraction solution (15 ml of 1% dH_2_O/acetic acid solution + 15 ml acetonitrile) to each tube and mixed thoroughly, followed by the addition of 6 g of anhydrous magnesium sulfate (MgSO_4_) and 1.5 g sodium acetate (NaAc). Tubes were mixed gently on an agitator for 10 min and then centrifuged. Fifteen millilitres of supernatant was removed and dispensed into a 15-ml Agilent dispersive Solid Phase Extraction tube containing 400 mg primary secondary amine, 400 mg C18 and 1,200 mg MgSO_4_. Two millilitres of toluene was added to each 15 ml Agilent tube, to aid in the extraction of planar pesticides from the pollen matrix^60^. Tubes were gently agitated, centrifuged and 200 μl of the supernatant from this final step of processing was transferred to 96-well plates for analysis by liquid chromatography (LC) and tandem mass spectrometry at the Bindley Bioscience Center at Purdue University. An Agilent Zorbax SB-Phenyl 4.6 × 150 mm, 5 μm column was used for LC separation (Agilent Technologies, Santa Clara, CA) and an Agilent 1200 Rapid Resolution LC system coupled to an Agilent 6460 series triple quadrupole mass spectrometer was used to identify pesticide residues based on retention time and co-chromatography with high-purity analytical standards of the 65 pesticide targets evaluated in our study (Table 7). Analytical-grade standards were purchased from Sigma-Aldrich, Accustandard and Fisher Scientific. We used deuterated internal standards to quantify the concentrations of the neonicotinoids acetamiprid, clothianidin, imidacloprid and thiamethoxam in pollen samples. Ten microlitres of a spiking solution containing only deuterated internal standards of these four neonicotinoids was added to 190 μl of each pollen sample for a total volume of 200 μl for analysis on the LC and tandem mass spectrometry instrument. A stock mixture of the 61 remaining analytical standards was created and from that mixture a series of 8 serial dilutions were conducted and analysed on the instrument to establish a standard curve to which pesticide residues in pollen samples was calibrated to determine final concentrations. The Agilent MassHunter METLIN Metabolite Personal Compound Database and Library^61^ was used to identify compounds based on known ratios of parent mass and at least two fragment transitions. The final concentration of pesticide residues was calculated by averaging the values detected from the three replicate 5 g pollen samples processed from each hive on each available date.

### Data availability

The data that support the findings of this study are available from the corresponding author on request.

## Additional information

**How to cite this article:** Long, E. Y. & Krupke, C. H. Non-cultivated plants present a season-long route of pesticide exposure for honey bees. *Nat. Commun.* 7:11629 doi: 10.1038/ncomms11629 (2016).

## Figures and Tables

**Table 1:** The mean, median and range of concentrations for 32 pesticide active ingredients detected in pollen samples collected from honey bee colonies over a 16-week period.

**Chemical**	**Pesticide type**	**Site 1: non-agricultural area**		**Site 2: untreated maize**		**Site 3: treated maize**		**LOD (p.p.b.)**
		**% of samples detected (*****n*****=30)**	**Mean, median conc. (p.p.b.)**	**% of samples detected (*****n*****=24)**	**Mean, median conc. (p.p.b.)**	**% of samples detected (*****n*****=32)**	**Mean, median conc. (p.p.b.)**	
Carbaryl	Carbamate	6.67%	<LOD, 0.0(0.12–0.30)	8.33%	0.07, 0.0(0.20–1.45)	25%	0.52, 0.0 (0.09–10.55)	0.02
Methomyl		6.67%	<LOD, 0.0 (0.12–0.15)	4.17%	0.09, 0.0 (2.07–2.07)	9.38%	<LOD, 0.0 (0.17–0.63)	0.09
Azoxystrobin	Fungicide	93.33%	1.63, 0.868 (0.1–10.39)	87.5%	1.90, 0.92 (0.10–8.32)	87.5%	2.39, 0.66 (0.09–28.31)	0.08
Difenoconazole		6.67%	0.39, 0.0 (4.65–6.93)	12.5%	0.21, 0.0 (0.54–2.73)	6.25%	<LOD, 0.0 (0.35–0.68)	0.14
Dimoxystrobin		20%	0.01, 0.0 (0.02–0.10)	8.33%	0.003, 0.0 (0.01–0.05)	ND	.	0.002
Metalaxyl		26.67%	0.09, 0.0 (0.03–1.45)	33.33%	0.09, 0.0 (0.03–0.68)	31.25%	0.05, 0.0 (0.03–0.42)	0.004
Propiconazole		23.33%	0.25, 0.0 (0.47–2.26)	37.5%	0.57, 0.0 (0.12–4.34)	21.88%	0.52, 0.0 (0.34–6.57)	0.05
Pyraclostrobin		36.67%	1.74, 0.0 (0.05–12.0)	33.33%	1.73, 0.0 (0.62–11.67)	28.13%	2.32, 0.0 (0.61–31.46)	0.04
Trifloxystrobin		63.33%	0.97, 0.156 (0.03–15.16)	62.5%	0.52, 0.16 (0.02–6.83)	65.63%	0.35, 0.11 (0.03–3.32)	0.02
2,4–D	Herbicide	ND	.	12.5%	<LOD, 0.0 (4.08–12.39)	ND	.	1.44
Acetochlor		10%	0.14, 0.0 (0.36–2.99)	25%	0.36, 0.0 (0.13–3.33)	34.38%	2.12, 0.0 (0.28–25.38)	0.12
Atrazine		20%	0.82, 0.0 (0.42–7.29)	54.17%	1.63, 0.43 (0.33–14.88)	43.75%	4.66, 0.0 (0.21–45.26)	0.02
Chlorimuron–ethyl		6.67%	0.30, 0.0 (3.51–5.35)	20.83%	0.25, 0.0 (0.46–1.97)	15.63%	0.11, 0.0 (0.20–1.38)	0.04
Metolachlor		83.33%	0.33, 0.13 (0.01–2.45)	75%	0.36, 0.20 (0.20–1.45)	71.88%	0.58, 0.11 (0.01–7.13)	0.003
DEET	Repellent	100%	3.82, 1.37 (0.10–21.12)	100%	2.48, 0.82 (0.08–20.33)	100%	2.91, 0.55 (0.07–26.20)	0.004
Methoxyfenozide	Insect growth regulator	3.33%	<LOD, 0.0 (0.16–0.16)	8.33%	0.06, 0.0 (0.48–0.91)	9.38%	0.35, 0.0 (0.42–10.25)	0.06
Spinetoram	Fermentation product insecticide	10%	<LOD, 0.0 (0.10–1.03)	16.67%	<LOD, 0.0 (0.13–0.46)	15.63%	<LOD, 0.0 (0.10–0.75)	0.08
Spinosad		6.67%	0.06, 0.0 (0.87 –1.01)	8.33%	0.05, 0.0 (0.57–0.64)	9.38%	0.03, 0.0 (0.16–0.44)	0.03
Acetamiprid	Neonicotinoid	ND	.	12.5%	0.04, 0.0 (0.07–0.65)	28.13%	0.32, 0.07 (0.04–4.69)	0.03
Clothianidin		3.33%	0.16, 0.0 (4.66–4.66)	16.67%	0.20, 0.0 (0.70–1.79)	21.88%	0.66, 0.0 (0.64–9.37)	0.09
Dinotefuran		10%	<LOD, 0.0 (4.85–6.31)	4.17%	<LOD, 0.0 (4.08–4.08)	3.13%	<LOD, 0.0 (4.52–4.52)	0.97
Imidacloprid		6.67%	<LOD, 0.0 (0.94–1.05)	ND	.	ND	.	0.69
Thiacloprid		ND	.	4.17%	<LOD, 0.0 (0.39–0.39)	3.13%	<LOD, 0.0 (0.25–0.25)	0.12
Thiamethoxam		10%	0.12, 0.0 (0.52–1.69)	33.33%	0.23, 0.0 (0.18–1.82)	21.88%	0.08, 0.0 (0.07–0.95)	0.04
Coumaphos	Organophosphate	16.67%	0.32, 0.0 (0.70–4.21)	20.83%	0.25, 0.0 (0.25–4.05)	9.38%	<LOD, 0.0 (0.26–1.44)	0.24
Diazinon		10%	0.13, 0.0 (0.15–2.22)	16.67%	0.08, 0.0 (0.20–0.74)	15.63%	0.04, 0.0 (0.08–0.43)	0.007
Dimethoate		3.33%	0.01, 0.0 (0.34–0.34)	20.83%	0.08, 0.0 (0.03–1.43)	15.63%	<LOD, 0.0 (0.12–0.14)	0.01
T-Fluvalinate	Pyrethroid	10%	<LOD, 0.0 (2.31–14.48)	4.17%	<LOD, 0.0 (5.88–5.88)	ND	.	1.13
Phenothrin		30%	47.6, 0.0 (34.92–343.76)	16.67%	84.5, 0.0 (250.39–728.85)	28.13%	195.4, 0.0 (274.13–1,955)	1.28
Prallethrin		46.67%	29.0, 0.0 (6.49–236.78)	25%	5.53, 0.0 (5.59–55.06)	15.63%	5.88, 0.0 (7.24–144.46)	0.68
Tetramethrin		6.67%	0.15, 0.0 (0.70–4.10)	8.33%	0.04, 0.0 (0.18–0.68)	6.25%	0.02, 0.0 (0.30–0.42)	0.02
λ-Cyhalothrin		10%	0.22, 0.0 (1.39–3.39)	16.67%	0.37, 0.0 (0.16–5.69)	12.5%	0.50, 0.0 (0.103–6.13)	0.005

DEET, N,N-Diethyl-meta-toluamide; LOD, limit of detection; ND, not detected.

Colonies were placed in a non-agricultural area, adjacent to a maize field grown from untreated seed and adjacent to a maize field grown from seed treated with neonicotinoids and fungicides.

**Table 2:** The identity and percentage of pollen collected by honey bees over a 16-week period.

**Plant family**	**% of pollen over 16 weeks**			**Plant genus/species**	**Common name**
	**Site 1**	**Site 2**	**Site 3**		
Fabaceae*	5.97	1.38	0.84	*G. max*	Soybean
	3.98	—	0.14	*Lotus corniculatus*	Common bird's-foot-trefoil
	6.91	0.59	0.52	*Melilotus* sp.	Sweet clover
	.	0.02	0.82	*Trifolium campestre*	Hop clover
	5.45	0.35	0.54	*Trifolium hybridum*	Alsike clover
	0.04	16.39	14.79	*Trifolium pratense*	Red clover
	2.02	15.84	10.35	*Trifolium repens*	White clover
	4.13	—	3.46	*Trifolium* sp.	Unknown clover
	8.24	1.18	—	—	NA
	—	0.02	—	—	Tufted vetch
Brassicaceae*	12.62	6.12	4.89	Unknown (likely including *S. arvensis)*	Mustards and cabbages
Asteraceae	0.83	8.3	6.8	*Ambrosia artemisiifolia*	Common ragweed
	.	0.02	0.02	*Arctium minus*	Wild rhubarb
	0.5	0.13	1.68	*Cichorium intybus*	Common chicory
	—	—	—	*Cirsium* sp.	Plume thistle
	—	—	—	*Helianthus* sp.	Sunflower
	2.21	1.31	0.88	*Solidago* sp.	Goldenrod
	—	0.04	0.07	*Taraxacum officinale*	Common dandelion
	0.08	3.19	3.76	Unknown	NA
Vitaceae	6.91	8	1.86	*Vitis* sp.	Wild grape
Plantaginaceae	6.66	7.14	10.99	*Plantago* sp.	Fleawort
Euphorbiaceae	0.1	6.6	9.67	*Mercurialis* sp.	Spurges
Rosaceae*	0.02	—	—	*Crataegus* sp.	Hawthorn
	—	—	—	*Malus* sp.	Apple
	0.04	–	—	—	Cinquefoils
	0.04	–	—	—	Plum, cherry, peach group
	3	0.2	—	—	Chokecherry
	0.06	0.02	0.14	*Rubus* sp.	Blackberry group
	3.48	0.09	.	Unknown	NA
Adoxaceae	6.49	3.34	6.03	*Sambucus* sp.	Elderberry
Araliaceae	4.92	—	—	*Aralia* sp.	Spikenard
Cyperaceae	3.42	0.04	—	*Carex* sp.	Sedges
Unknown (P7A-50 #4)	0.17	5.96	4.26	Unknown	NA
Poaceae*	0.06	2.77	3.87	*Zea mays*	Maize
Umbelliferae*	3.07	1.22	2.27	Unknown	Carrot, celery, parsley group
Caprifoliaceae	2.8	0.02	0.02	*Lonicera* sp.	Honeysuckle
	—	0.04	0.16	*Diervilla* sp.	Bush honeysuckle
Unknown (1-5 apertures)	1.15	0.79	0.73	Unknown	NA
Oxalidaceae	0.04	2.56	0.02	*Oxalis* sp.	Wood sorrel
Hypericaceae	1.04	0.22	0.82	*Hypericum* sp.	St. John's wort
Liliaceae	0.54	1.7	0.41	Unknown	Lily family
Anacardiaceae	0.13	0.81	2.02	*Rhus typhina*	Staghorn sumac
Ranunculaceae	0.98	—	—	—	Columbine
	—	0.02	—	—	NA
Cornaceae	0.06	0.92	0.73	*Cornus stolonifera*	Red-osier dogwood
Salicaceae	0.02	0.68	0.07	*Populus* sp.	Poplar, aspen, cottonwood
	0.1	—	1.7	*Salix* sp.	Willow
Rhamnaceae	0.02	0.02	1.5	*Rhamnus* sp.	Buckthorn
Amaranthaceae	0.65	0.35	0.18	*Chenopodium* sp.	Goosefoot
Gramineae	0.38	0.31	—	Unknown	True grasses
Malvaceae	0.31	—	0.02	*Tilia* sp.	Basswood
Balsaminaceae	0.25	0.15	0.2	*Impatiens capensis*	Common jewelweed
Unknown (P7A-16 #4)	0.08	—	—	Unknown	NA
Betulaceae	—	0.35	0.27	*Corylus* sp.	Hazel
Caryophyllaceae	—	0.31	—	Unknown	Carnation family
Unknown (P7A-29 #6)	—	0.22	—	Unknown	NA
Unknown (P7A-28 #7)	—	0.17	—	Unknown	NA
Unknown (P7A-47 #9)	—	0.09	0.2	Unknown	NA
Unknown (P7A-47 #8)	—	—	0.2	Unknown	NA
Unknown (P7A-41 #14)	—	—	0.11	Unknown	NA
Nymphaeaceae	—	—	0.07	*Nuphar* sp.	Water lily
Polygonaceae	—	—	0.05	*Rumex* sp.	Knotweed

Honey bee hives were placed at one of three sites: (1) in a non-agricultural area (*n*=28 plant families), (2) adjacent to a maize field grown from untreated seeds (*n*=30 plant families) or (3) adjacent to a maize field grown from pesticide-treated seeds (*n*=29 plant families). Note: Asterisks denote plant families that do or may include cultivated plant species. Dashes denote that pollen from a particular plant family was not observed in honey bee pollen loads.

**Table 3:** The average risk (%) posed by pesticide residues in pollen collected by honey bees placed in a non-agricultural area.

**Date**	**Active Ingredient (AI)**										**Synergies**	**Total Weekly Risk**
	**Clothianidin**	**Coumaphos**	**Diazinon**	**Dinotefuran**	**Imidacloprid**	**Phenothrin**	**Prallethrin**	**Tetramethrin**	**Thiamethoxam**	**λ-cyhalothrin**	**Propiconazole+λ -cyhalothrin**	
Use*	ST	DP, S	DP, G, LC	G, S	C, G, D, ST	D, S	S	S	ST	C, D	C, D	
24-May	—	—	—	—	—	—	—	—	—	—	—	0
31-May	—	C: 0.02	C: 0.03	—	O: 0.02C: 0.05	C: 6.1	C: 184	C: 0.09	O: 0.03C: 0.13	—	—	O: 0.05C: 191
7-Jun	O: 0.13C: 0.19	C: 0.01	—	—	—	—	—	—	O: 0.11C: 0.42	O: 0.04C: 0.34	C: 13.5	O: 0.28C: 14.4
14-Jun	—	—	—	—	—	—	C: 171	—	—	O: 0.02C: 0.14	C: 5.5	O: 0.02C: 177
20-Jun	—	—	—	—	—	—	—	—	—	—	—	0
27-Jun	—	—	—	O: 0.08C: 0.48	—	—	C: 9.0	—	—	—	—	O: 0.08C: 9.5
5-Jul	—	—	—	—	—	—	C: 81.3	—	—	O: 0.02C: 0.18	C: 6.9	O: 0.02C: 88.4
12-Jul	—	C: 0.01	C: 0.02	O: 0.08C: 0.48	O: 0.02C: 0.06		C: 27.7	C: 0.01	O: 0.10C: 0.37	—	—	O: 0.20C: 28.7
19-Jul	—	—	—	—	—	—	C: 5.0	—	—	—	—	O: 0C: 5.0
26-Jul	—	—	—	—	—	—	C: 16.2	—	—	—	—	O: 0C: 16.2
2-Aug	—	—	—	—	—	—	C: 78.8	—	—	—	—	O: 0C: 78.8
9-Aug	—	—	—	—	—	—	C: 66.9	—	—	—	—	O: 0C: 66.9
16-Aug	—	—	—	—	—	C: 34.8	C: 17.2	—	—	—	—	O: 0C: 52.0
23-Aug	—	—	—	O: 0.10C: 0.63	—	C: 20.2		—	—	—	—	O: 0.10C: 20.8
30-Aug	—	—	—	—	—	C: 61.2	C: 18.6	—	—	—	—	O: 0C: 79.7
13-Sep	—	—	—	—	—	C: 42.2	—	—	—	—	—	O: 0C: 42.2
Total seasonal risk/AI	*0.32*	0.04	0.05	*1.85*	*0.15*	*164.5*	*675.7*	*0.10*	*1.16*	*0.74*	*25.9*	

Oral (O) and contact (C) risks are based on the residue load, frequency of detection, and reported individual or synergistic lethal doses (LD_50_) of active ingredients. Low risk values: 0.1–1.0; Moderate risk values: 1–5; High risk values: >5. Risk values < 0.01 not shown. Note: Dashes denote no detection of active ingredients. Italicized values denote relevant seasonal risk values for honey bees.

^*^C, liquid, soluble, or emulsifiable concentrate; D, dusts or dustable powder; G, granules; S, spray; SD, soil drench; ST, seed treatment.

**Table 4:** The average risk (%) posed by pesticide residues in pollen collected by honey bees placed next to an untreated maize field.

**Date**	**Active ingredient (AI)**								**Synergies**			**Total weekly risk**
	**Clothianidin**	**Diazinon**	**Dinotefuran**	**Phenothrin**	**Prallethrin**	**Tetramethrin**	**Thiamethoxam**	**λ-cyhalothrin**	**Propiconazole+Acetamiprid**	**Propiconazole+Thiacloprid**	**Propiconazole+λ-cyhalothrin**	
Use*	ST	DP, G, LC	G, S	D, S	S	S	ST	C, D	D, S	C, G, S	C, D	
24 May	—	—	—	—	—	—	—	—	—	—	—	0
31 May	O: 0.22C: 0.34	—	—	—	C: 11.9	—	O: 0.26C: 1.00	—	—	—	—	O: 0.48C: 13.24
7 June	—	—	—	—	C: 2.64	—	O: 0.47C: 1.82	O: 0.06C: 0.47	—	—	C: 18	O: 0.53C: 22.93
14 June	O: 0.24C: 0.37	—	—	—	C: 22.9	—	O: 0.28C: 1.06	O: 0.11C: 0.95	—	—	C: 36.7	O: 0.63C: 61.98
20 June	—	—	—	—	C: 2.33	—	O: 0.04C: 0.15	C: 0.03	—	—	C: 1.19	O: 0.04C: 3.7
27 Jun	—	C: 0.03	—	—	—	C: 0.02	—	—	—	—	—	C: 0.05
5 July	O: 0.09C: 0.14	—	—	—	—	—	—	—	—	—	—	O: 0.09C: 0.14
12 July	—	—	—	—	—	—	O: 0.09C: 0.35	C: 0.03	C: 0.21	—	C: 1.00	O: 0.09C: 1.59
19 July	—	—	O: 0.03C: 0.17	—	—	—	—	—	C: 0.06	O: 0.02C: 0.12	—	O: 0.05C: 0.35
26 July	—	—	—	—	—	—	O: 0.04C: 0.16	—	—	—	—	O: 0.04C: 0.16
2 August	—	—	—	—	—	—	—	—	—	—	—	0
9 August	O: 0.20C: 0.31	—	—	—	—	—	—	—	—	—	—	O: 0.20C: 0.31
16 August	—	—	—	C: 91.3	C: 17.6	—	—	—	—	—	—	C: 108.9
23 August	—	—	—	C: 32.1	—	—	—	—	—	—	—	C: 32.1
30 August	—	—	—	C: 42.3	C: 13.4	—	—	—	—	—	—	C: 55.7
13 September	—	—	—	C: 93.5	—	—	—	—	—	—	—	C: 93.5
Total seasonal risk/AI	*1.91*	0.03	*0.20*	*259.2*	*70.77*	0.02	*5.72*	*1.65*	*0.27*	*0.14*	*56.89*	

Oral (O) and contact (C) risks are based on the residue load, frequency of detection and reported individual or synergistic lethal doses (LD_50_) of active ingredients. Low risk values: 0.1–1.0; moderate risk values: 1–5; high risk values: > 5. Risk values <0.01 not shown. Dashes denote no detection of active ingredients. Italicized values denote relevant seasonal risk values for honey bees.

^*^C, liquid, soluble, or emulsifiable concentrate; D, dusts or dustable powder; G, granules; S, spray; SD, soil drench; ST, seed treatment.

**Table 5:** The average risk (%) posed by pesticide residues in pollen collected by honey bees placed next to a pesticide-treated maize field.

**Date**	**Active ingredient (AI)**									**Synergies**			**Total weekly risk**
	**Acetamiprid**	**Carbaryl**	**Clothianidin**	**Diazinon**	**Dinotefuran**	**Phenothrin**	**Prallethrin**	**Thiamethoxam**	**λ-cyhalothrin**	**Propiconazole+Acetamiprid**	**Propiconazole+Thiacloprid**	**Propiconazole+λ-cyhalothrin**	
Use*	S, SD	C, D, G, S	ST	DP, G, LC	G, S	D, S	S	ST	C, D	D, S	C, G, S	C, D	
24 May	—	O: 0.01C: 0.03	O: 0.11C: 0.18	—	—	—	—	O: 0.13C: 0.52	—	—	—	—	O: 0.25C: 0.73
31 May	—	—	—	—	—	—	—	—	—	—	—	—	0
7 June	—	—	—	—	—	—	C: 2.88	C: 0.04	O: 0.15C: 1.23	—	—	C: 40.4	O: 0.15C: 44.55
14 June	—	—	—	—	—	—	C: 37.6	—	O: 0.09C: 0.77	C: 0.01	—	C: 25.1	O: 0.09C: 63.48
20 June	—	C: 0.02	O: 2.37C: 3.65	—	—	—	—	C: 0.05	—	—	—	—	O: 2.37C: 3.72
27 June	—	—	—	C: 0.01	—	—	—	O: 0.02C: 0.39	C: 0.01	—	—	C: 0.42	O: 0.02C: 0.83
5 July	—	—	—	—	—	—	—	—	—	—	—	—	0
12 July	C: 0.01	O: 0.07C: 0.19	—	—	O: 0.02C: 0.14	—	—	C: 0.11	—	C: 2.86	—	—	O: 0.09C: 3.31
19 July	—	—	—	—	—	—	C: 1.89	—	—	C: 0.18	—	—	C: 2.07
26 July	—	—	—	—	—	—	—	—	—	—	—	—	0
2 August	—	—	O: 0.15C: 0.24	—	—	—	—	—	—	—	—	—	O: 0.15C: 0.24
9 August	—	—	—	—	—	C: 42.4	C: 2.94	—	—	C: 0.01	—	—	C: 45.35
16 August	—	—	O: 0.58C: 0.89	—	—	C: 82.3	C: 3.73	—	—	C: 0.01	—	—	O: 0.58C: 86.93
23 August	—	—	O: 0.52C: 0.80	—	—	C: 142.2	—	C: 0.10	—	C: 0.18	—	—	O: 0.52C: 143.28
30 August	—	—	—	C: 0.01	—	C: 95.1	—	O: 0.10C: 0.20	—	C: 0.17	C: 0.05	—	O: 0.01C: 95.53
13 September	—	—	—	—	—	C: 314.3	—	—	—	C: 0.02	—	—	C: 314.32
Total seasonal risk/AI	0.01	*0.32*	*9.49*	0.02	*0.16*	*676.3*	*49.04*	*1.57*	*2.25*	*3.44*	0.05	*65.92*	

Oral (O) and contact (C) risks are based on the residue load, frequency of detection, and reported individual or synergistic lethal doses (LD_50_) of active ingredients. Low risk values: 0.1–1.0; moderate risk values: 1–5; high risk values: >5. Risk values <0.01 not shown. Dashes denote no detection of active ingredients. Italicized values denote relevant seasonal risk values for honey bees.

^*^C, liquid, soluble, or emulsifiable concentrate; D, dusts, dustable powder, or wettable powder; G, granules; S, spray; SD, soil drench; ST, seed treatment.

**Table 6:** Proportional land use types in a 2 kilometer radius surrounding the locations where honey bee colonies were placed in 2011.

**USDA-NASS label**	**Site 1: non-agricultural area**	**Site 2: untreated maize**	**Site 3: treated maize**
Maize	10.5%	31.8%	37.1%
Soybean	1.8%	40.0%	36.8%
Winter wheat	0.22%	0.96%	1.7%
Alfalfa, hay, pasture	13.4%	16.1%	12.3%
Open water	5.0%	0%	0%
Developed	46.6%	5.9%	6.2%
Deciduous forest	21.4%	5.0%	5.7%
Other*	1.0%	0.16%	0.13%

^*^Land use types that each comprised < 1% of the area within a 2 km radius of each site (thatis, woody or herbaceous wetlands, barren land, tomatoes, or shrubland).

**Table 7:** List of 65 pesticides screened in honey bee pollen over a 16-week period

**Chemical name**	**Pesticide type**
Aldicarb	Carbamate insecticide
Carbaryl	
Methomyl	
Spinosad	Fermentation product insecticide
Spinetoram	
Azoxystrobin	Fungicide
Captan	
Chlorothalonil	
Tetrahydropthalimide	
Difenoconazole	
Dimoxystrobin	
Fenbuconazole	
Metalaxyl	
Propiconazole	
Prothioconazole	
Pyraclostrobin	
Trifloxystrobin	
2,4-D	Herbicide
Acetochlor	
Alachlor	
Atrazine	
Chlorimuron-ethyl	
Flumioxazin	
Metolachlor	
Methoxyfenozide	Insect growth regulator
Acetamiprid	Neonicotinoid
Clothianidin	
Dinotefuran	
Imidacloprid	
Nitenpyram	
Thiacloprid	
Thiamethoxam	
Chlorpyrifos	Organophosphate insecticide
Coumaphos	
Demeton	
Diazinon	
Dimethoate	
Malathion	
Methidathion	Organophosphate insecticide
Phosmet	
Phoxim	
Terbufos	
Trichloronate	
β-Cyfluthrin	Pyrethroid insecticide
λ-Cyhalothrin	
Allethrin	
Bifenthrin	
Cyhalothrin	
Cypermethrin	
Deltamethrin	
Esfenvalerate	
Fenpropathrin	
Fluvalinate	
Permethrin	
Phenothrin	
Prallethrin	
Tetramethrin	
DEET	Repellent
Fipronil	Phenylpyrazole insecticide
Indoxacarb	Oxadiazine insecticide
Endosulfan sulfate	Organochlorine insecticide and acaricide
α-Endosulfan	
β-Endosulfan	
DMFP	Acaricide
Propargite	

DEET, N, N-Diethyl-meta-toluamide; DMFP, N-2,4-dimethylphenyl-N-methylformamidine.
